# Unusual Fatigue Crack Growth Behavior of Long Cracks at Low Stress Intensity Factor Ranges

**DOI:** 10.3390/ma17040792

**Published:** 2024-02-06

**Authors:** Daniel Kujawski, Asuri K. Vasudevan

**Affiliations:** 1 Mechanical and Aerospace Engineering, Western Michigan University, Kalamazoo, MI 49008, USA; 2 TDA Inc., Falls Church, VA 22043, USA

**Keywords:** FCG threshold, vanishing of threshold, FCG below-threshold, Marci effect

## Abstract

In this article, we characterize and review the unusual lack of threshold in fatigue crack growth (FCG) behavior for some alloys at low values of stress intensity factor ranges ΔK and its implications to damage-tolerant design approaches. This unusual behavior was first observed by Marci in 1996 in IMI 834 alloy. Conventional applications of linear elastic fracture mechanics to FCG analysis at constant R-ratio (or K_max_) assumes that (da/dN) decreases monotonically with decreasing ΔK and approaches the threshold value of ΔK_th_ with (da/dN) ≤ 10^−7^ mm/cycle for a given R (or K_max_). However, instead of ΔK threshold behavior, some materials exhibit plateau or acceleration in da/dN rate with decreasing ΔK for long cracks tested in both constant R and K_max_ conditions. This unusual (da/dN)-ΔK behavior is only observed experimentally but not understood and represents a challenge to scientists and engineers to model the safe fatigue life prediction of structures under low amplitude vibrating loads.

## 1. Introduction

**Figure 1:**
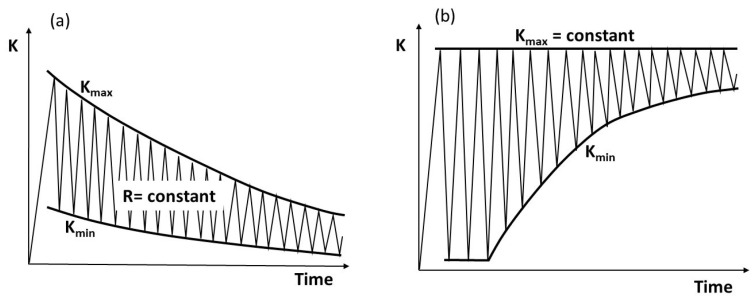
Illustration of (**a**) R-constant and (**b**) K_max_ = constant tests.

Traditionally, the fatigue crack growth (FCG) behavior of materials is researched using fracture mechanics specimens [1,2], tested at a constant load ratio R (=K_min_/K_max_) or constant K_max_ as illustrated in Figure 1. In the early 1960s, Paris and Erdogan [3] proposed correlating FCG rate, (da/dN), versus the applied range of stress intensity factor, ΔK, as follows:

(1) $$\frac{da}{dN}=C{\Delta K}^{m}$$

where C and m are curve fitting parameters. It can be noted that in general, it is widely accepted that FCG is a two-parameter process involving ΔK and K_max_ (or R). In the case of R = 0, Equation (1) reduces to ΔK only, since ΔK = K_max_.

**Figure 2:**
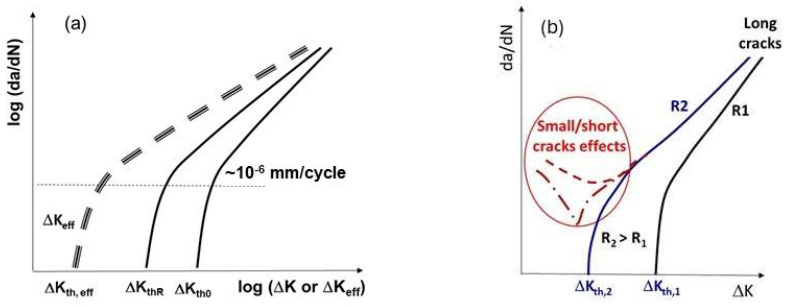
Illustration of FCG (da/dN) vs. ΔK (**a**) indicating ΔK_th_ with monotonically decreasing (da/dN) with decreasing ΔK (or ΔK_eff_), (**b**) showing unusual behavior of small/short cracks.

The relationship shown in Equation (1) is commonly known as the Paris law. Later, it was discovered that around (da/dN) $\sim 10^{-6}$ mm/cycle the actual (da/dN) curve starts to deviate from Equation (1) as ΔK decreases towards the threshold ΔK_th_ (Figure 2a).

The threshold ΔK_th_ is usually defined as (da/dN) ≤ 10^−7^ mm/cycle [1,2], which is in the range of the lattice parameter of the material. For tests conducted with constant R-ratios, different values of ΔK_th_ are observed as (da/dN) decreasing monotonically with decreasing ΔK. Typically, at high values of R (>0.6–0.7) the dependence of ΔK_th_ on R vanishes, approaching a steady value. This is traditionally attributed to the absence of crack closure at high R-ratios. Usually, smaller variations of ΔK_th_ are observed when tests are conducted with K_max_ = constant, often believed to be due to closure free FCG for K_max_ tests. Conventional applications of linear elastic fracture mechanics to FCG analysis at constant R-ratio assumes that (da/dN) decreases monotonically with decreasing ΔK and approaches the threshold value of ΔK_th_ with (da/dN) ≤ 10^−7^ mm/cycle for a given R. 

To account for R-ratio effects in (da/dN) and ΔK_th_, Elber [4] proposed to plot (da/dN) vs. ΔK_eff_ = K_max_ − K_cl_, where K_cl_ is the stress intensity factor corresponding to crack closure. The idea behind ΔK_eff_ was to collapse all the (da/dN) curves (and ΔK_th_) for different R-ratios into one common “effective curve”, which can be used in design. The most referred mechanism of crack closure, proposed initially by Elber, is plasticity-induced crack closure (PICC). Later, due to limited plasticity at the near-threshold and inadequacy of PICC to correlate ΔK_th_ with R, other mechanisms such as oxide-induced and roughness-induced crack closure (OICC and RICC) were suggested to account for R-ratio effects on threshold and near-threshold FCG behavior [5,6]. Recently, we have reviewed and re-examined the effects on these closure mechanisms and their inability to shield the crack tip from the applied load [7] for the observed R-ratio effects on FCG in the air or in an active environment rather than crack closure, since in vacuum R-ratio effect on FCG disappears or is insignificant. Knowing the value of ΔK_th_ at a given R (or ΔK_th,eff_), designers assume that below these thresholds a long crack does not propagate in practical applications. Such an assumption requires the existence of ΔK_th_.

In contrast, it is observed experimentally that small/short cracks do not exhibit threshold behavior [5] and may propagate below ΔK_th_ for long cracks as illustrated in Figure 2b. Such behavior is usually treated as an “unusual” trend of small/short cracks by asserting that they are free from PICC due to the lack of substantial crack wake plasticity. Then, as small/short cracks grow longer, the associated wake and PICC develop, and they approach long cracks behavior (Figure 2b). This explanation for PICC has been debated for the last 50 years [7], since at near-threshold, the crack tip plasticity is rather limited. Hence, instead of PICC, other mechanisms such as OICC and RICC are often explored. 

On the other hand, there are many examples published in the literature, e.g., [8,9,10,11,12,13,14,15,16], over the last 30 years, regarding an “unusual” FCG of long cracks at low ΔK below the ΔK_th_. Such unusual long crack behavior is manifested by the lack of traditional threshold in both K_max_ and R = constant tests. This experimentally observed lack of ΔK_th_ for long cracks was mostly ignored or overlooked in mainstream research as unanticipated anomalies despite its critical importance in the safe design of structures using fracture mechanics methodology. Based on the current understanding, design engineers take advantage of the FCG threshold concept, which states that if the ΔK applied is below ΔK_th_ no significant FCG occurs. However, if cracks grow with (da/dN) > 10^−7^ mm/cycle at the applied ΔK < ΔK_th_ and do not reach ΔK_th_, then the traditional design approach based on the long crack threshold ΔK_th_ may be nonconservative and unsafe. 

In this article, the unusual FCG behavior of long cracks at low stress intensity factor ranges ΔK (below conventional ΔK_th_), as documented in the literature, is reviewed. This review draws upon results from several alloys, loading conditions and specimen geometries to illustrate this unexpected FCG phenomenon. This unusual (da/dN)-ΔK behavior is not fully understood and cannot be explained based on crack closure. The awareness and better understanding of this unusual behavior presents a challenge to scientists and engineers. Further ignoring or overlooking this phenomenon at low ΔK may compromise safety and lead to nonconservative fatigue life prediction of structures under low amplitude vibrating loads.

## 2. Closure and Two-Parameter Characterization of FCG Threshold

Since the 1970s, the fatigue community has focused on a single parameter, ΔK_eff_ (=K_max_ − K_cl_), as the driving force for fatigue crack growth (FCG) analysis [1,2,3,4]. ΔK_eff_ is commonly estimated from the deviation in the non-linearity (defined as K_cl_) in the load–displacement curve using a cracked sample. A long crack will not propagate if the following is applied: ΔK_eff_ < ΔK_eff,th_. Despite more than 50 years of research, there are still unresolved experimental challenges related to the determination of the ΔK_eff_ at threshold [7].

The first analytical assessment on the effect of crack flanks contact (or closure) using dislocation analysis was given by Vasudevan et al. in 1992 [17]. They demonstrated that premature crack flanks contact caused by the presence of oxides/asperities does exist. However, the magnitude of the closure contributions to the crack tip shielding is approximately 0.25 K_cl_ as determined from the experimental load–displacement measurements. Five years later, in 1997, a similar dislocation analysis was conducted by Riemelmoser and Pippan [18] for a linear elastic crack with a rigid single asperity and multiple asperities. Their analysis revealed that the contribution from a single asperity contact is only about 0.21 K_cl_. This finding was consistent with the conclusion drawn earlier by Vasudevan et al.’s [17] analysis where the shielding was determined to be 0.25 K_cl_. Recently, Pippan and Hohenwarter [6] have provided a comprehensive review on the PICC, OICC and RICC phenomena. By considering multiple rigid asperities contacting the crack flanks at the same K_cl_ (similar to a rigid wedge), the calculated crack tip shielding contribution was about 0.7 K_cl_, closely aligning with Paris et al.’s [19] analysis of partial crack closure (a rigid wedge at a small distance from the crack tip) indicated (2/π) K_cl_ = 0.64 K_cl_. Depending on the contact types (single or multiple rigid asperities), the shielding effect varied from 0.21 to 0.7 of K_cl_. It is worth noting that roughness-induced asperities are not rigid but exhibit elastic–plastic behavior since they are ductile and deformable. Therefore, this may significantly reduce the actual effect of theoretically calculated crack tip shielding.

In the 1990s, Vasudevan and Sadananda proposed that an unambiguous description of fatigue crack growth demands two stress intensity factor (SIF) parameters, namely ΔK and K_max_ [20]. By plotting ΔK vs. K_max_ for a given da/dN = constant, so called L-shaped curves are obtained. These L-shaped curves represent a material response in terms of a constant da/dN rate to the applied ΔK and K_max_.

**Figure 3:**
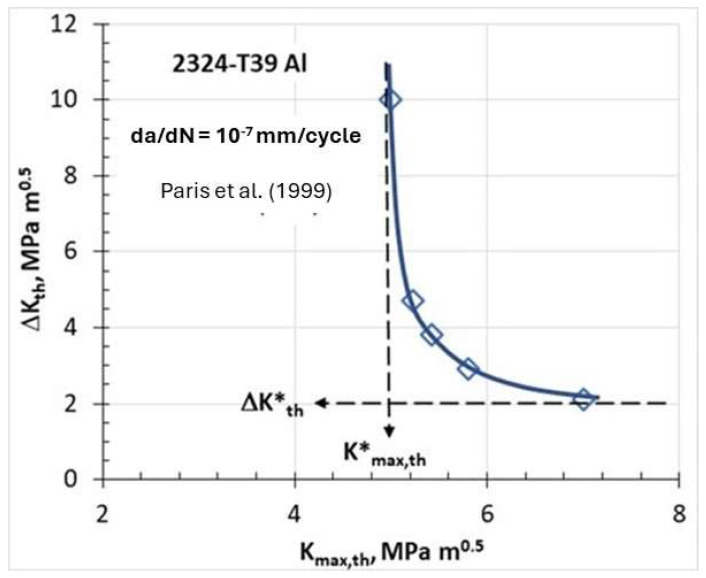
FCG threshold representation in terms of two parameters [19].

According to the ASTM standard E647 [1], the threshold may be defined when the FCG rate da/dN is less than or equal to 10^−7^ mm/cycle. By plotting the applied ΔK_th_ and K_max,th_ corresponding to da/dN = 10^−7^ mm/cycle the threshold region L-shaped curve is obtained as shown in Figure 3.

The significance of the threshold L-shaped curve means that the threshold comprises not one but two fracture mechanics parameters ΔK_th_ and K_max,th_. The threshold L-shaped curve defines the mutual interrelation between ΔK_th_ and K_max,th_ for any R-ratio to sustain da/dN = 10^−7^ mm/cycle. From the asymptotic nature of the L-shaped curve, two limiting values of ΔK*_th_ and K*_max,th_ can be determined. Any point on the L-shaped curve corresponding to da/dN = 10^−7^ mm/cycle satisfies both limiting values ΔK*_th_ and K*_max,th_ simultaneously, i.e., ΔK_th_ ≥ ΔK*_th_ and K_max,th_ ≥ K*_max,th_. If only one of these limiting values is satisfied, the crack should be arrested and not propagate. Vallellano et al. [21] provided a micromechanical description of two thresholds for FCG based on the successive blocking of both the monotonic plastic zone (MPZ) and the cyclic plastic zone (CPZ) of a fatigue crack by microstructural barriers, such as grain boundaries. This microstructural model establishes two thresholds: K*_max,th_ related to MPZ and ΔK*_th_ related to CPZ when they are blocked by grain boundaries. Both macro descriptions, in terms of limiting K*_max,th_ and ΔK*_th_, proposed by Vasudevan et al. [20], and the microstructural description proposed, by Vallellano et al. [21], in terms of the blockage of MPZ and CPZ, are consistent with experimental observations and logically coherent. Hence, the area on the right from the dashed vertical line, corresponding to K*_max,th_ (Figure 3), and below the dashed horizontal line, corresponding to ΔK*_th_, represents loading conditions with high K_max_ > K*_max,th_ and low ΔK < ΔK*_th_; therefore, the long crack should not propagate since only one threshold condition is satisfied, i.e., K_max_ > K*_max,th_. This type of fatigue loading is associated with high mean load and small cyclic oscillations as seen in high-speed rotating equipment, e.g., turbine blades or hafts. The “Marci effect” is associated with the loading regime where only one threshold is satisfied, namely when the applied K_max_ is larger than K*_max,th_ whereas the applied ΔK is smaller than ΔK*_th_. The next section describes this unusual FCG behavior observed experimentally and reported in the literature, which cannot be explained by crack closure or two-parameter threshold descriptions.

## 3. “Unusual” FCG Behavior Reported in the Literature

The following section characterizes the experimentally observed unusual FCG behavior of long cracks at low ΔK applied in several Ti superalloys, Al alloys and steel. It was also shown that some Ni-based superalloys, such as Ni100, Inconel 718 and Rene’95, do not seem to exhibit this unusual behavior.

### 3.1. Observations in Ti-Superalloys

**Figure 4:**
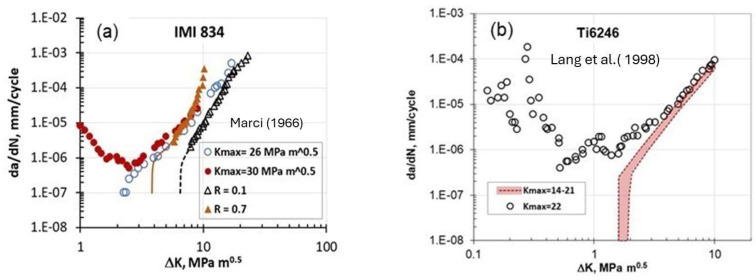
An accelerated FCG at low ΔK values for (**a**) IMI 834 alloy [8], and (**b**) Ti6246 superalloy [9] at different K-constant tests.

Marci [8], in 1996, was the first to observe experimentally that under a certain K_max_ constant test a long crack exhibits FCG rate at ΔK< ΔK_th_ akin to small/short cracks. The tests at a constant K_max_ were conducted at a frequency of 50 Hz using compact tension (CT) specimens. These specimens, with a thickness of 10 mm and a width of 50 mm, were machined from an IMI 834 forged disk. The crack length was measured using a DC potential drop method. The plane strain fracture toughness, K_IC_, for the investigated IMI 834 alloy ranged from 38 to 42 MPa$\sqrt{m}$, where a higher value of K_IC_ was found for samples taken from the mid-thickness of the forged disk. Figure 4a illustrates such an unusual behavior of long cracks data for the IMI 834 titanium superalloy [8] and contrasted this behavior with typical data for R = constant tests. Figure 4a indicates that this behavior begins at FCG rates corresponding to the knee point between the near-threshold and the Paris region (~10^−6^ mm/cycle). It seems that there is a controlling K_max_ value larger than 26 MPa$\sqrt{m}$ (just above 60% of K_IC_) when such unusual FCG originates. Below this controlling K_max_, a typical monotonic decrease in (da/dN) is observed as ΔK is reduced. At present, a consistent model, and a plausible explanation of this FCG behavior called the “Marci effect” does not exist.

After Marci’s paper [8], two years later in 1998, Lang et al. [9] were the first who investigated the “Marci effect” using nickel Ni-100 and titanium Ti-6246 superalloys under different K_max_ constant tests. Standard compact tension (CT) specimens with a width of 40 mm and a thickness of 10 mm were employed for the K_max_ constant tests, which were controlled by the MATE computer software. The crack length was monitored using the DC potential difference measurement (PDM) technique and a frequency of 50 Hz was used during the tests. The threshold crack growth was determined by the criterion da/dN < 10^−7^ mm/cycle.

They concluded that the Marci effect was absent in the Ni-100 alloy. In the case of the Ti-6246 alloy, the Marci effect occurred for K_max_ constant tests conducted with K_max_ ≥ 22 MPa$\sqrt{m}$, as shown in Figure 4b (K_max_ = 22 MPa$\sqrt{m}$ is about 72% of K_IC_). The influence of room temperature creep seems to be low since no change was observed at the two frequencies, 50 Hz and 10 Hz.

Shortly after Lang et al.’s [9] publication, Petit and his group [10] presented at the 2000 Conference in Cracow, Poland, their investigation on FCG behavior in the Ti-6246 superalloy in lab air and under vacuum (10^−4^ Pa) using K_max_ constant tests. Fatigue crack growth experiments were carried out on CT specimens (10 mm thick and 40 mm wide). The specimens underwent cyclic sinusoidal loading at a frequency of 35 Hz. The tests at constant K_max_ were conducted in conditions where K_min_ was higher than the stress intensity level for crack closure so to eliminate closure in all K_max_ constant tests.

**Figure 5:**
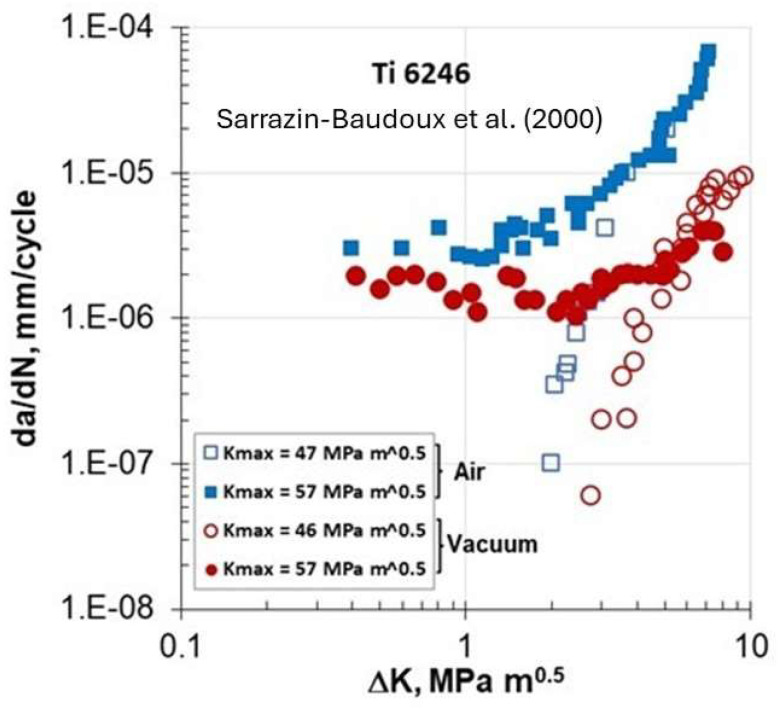
FCG of Ti 6246 alloy in lab air and under vacuum at different K_max_ constant tests [10].

Figure 5 shows their results for two K_max_ levels of 46–47 MPa$\sqrt{m}$ and 57 MPa$\sqrt{m}$. At 46–47 MPa$\sqrt{m}$, typical threshold behavior was observed in air and under vacuum, respectively. On the other hand, at K_max_ = 57 MPa$\sqrt{m}$, the Marci effect behavior was seen in both environments at low ΔK < ΔK_th_ ≅ 2 MPa$\sqrt{m}$. The authors concluded that since this behavior was present in both environments, this suggests that it was an ‘intrinsic’ mechanism. Hence, the Marci effect (da/dN) of FCG in air is about three times faster than under vacuum; this indicates that some environmental factors assist the cracking process in lab air. They also conducted tests at 57 MPa$\sqrt{m}$ at two frequencies, 3.5 Hz and 35 Hz. For ΔK ≥ 3 MPa$\sqrt{m}$, no frequency effect on da/dN was observed but for ΔK < 2 MPa$\sqrt{m}$, the FCG rate was faster at 3.5 Hz than at 35 Hz. The same two frequency data (not shown here) when plotted in terms of (da/dt) indicated that the frequency effect had almost vanished. They concluded that the FCG behavior for ΔK < 2 MPa$\sqrt{m}$ was a time-dependent process. Their observation possibly leads to a creep effect.

### 3.2. Observations in Al Alloys

**Figure 6:**
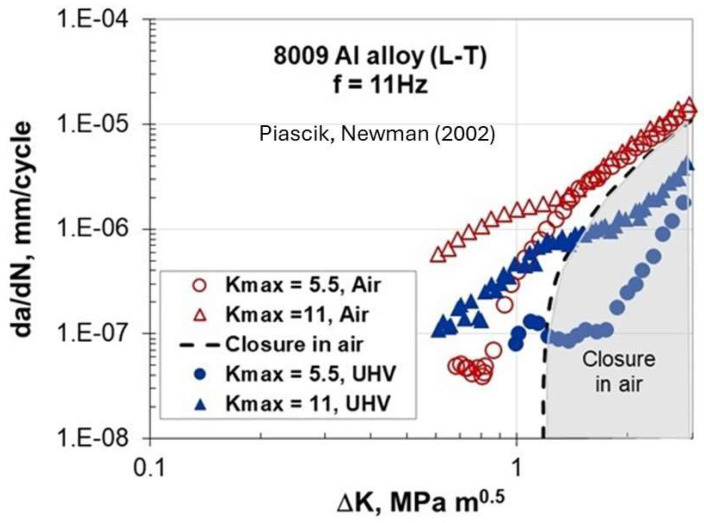
Near-threshold data for K_max_ constant tests at 5.5 and 11 MPa$\sqrt{m}$ in lab air and under ultra-high vacuum (UHV) [11]. Transition to accelerated FCG rates was associated with slant crack growth.

Figure 6 shows the FCG data for the 8009 Al alloy studied by Piascik and Newman [11] in lab air and under ultra-high vacuum (UHV ~10^−6^ Pa). The tests were conducted using ASTM eccentrically loaded single-edge-notch tension ESE(T) specimens with a width of 31.1 mm and a thickness of 2.3 mm. Continuous monitoring of crack length was carried out using the back-face strain compliance method. In the constant K_max_ test, K_min_ (and R) was increased to reduce ΔK. This test procedure led to high values of R in the near-threshold regime, thus eliminating the effects of crack closure.

Near-threshold data were obtained for two K_max_ constant tests at 5.5 and 11 MPa$\sqrt{m}$. A change in the (da/dN) slope is seen in Figure 6 for the 8009 alloy at low ΔK outside the closure region, where FCG rates in air are about five times faster than in the UHV, suggesting that the environment has an effect on the crack propagation rate. This accelerated FCG behavior indicated an increase in void production along the crack faces, which was limited to the specimen interior (plane–strain conditions), promoting crack tunneling. At certain K_max_, a transition to slant cracking occurred at threshold, which promoted an accelerated FCG rate with decreasing ΔK. Additional tests conducted at different frequencies, when plotted in terms of (da/dt), with an abnormal FCG indicated that the frequency effect almost vanished. They concluded that the peculiar FCG behavior at low ΔK was associated with the time-dependent process.

In addition to the 8009 Al alloy, Newman and Piascik [12] conducted near-threshold FCG of fine-grain nickel-based superalloys, Inconel 718 and Rene’95, and similar to Lang et al.’s [9] observation, the Marci effect was absent.

**Figure 7:**
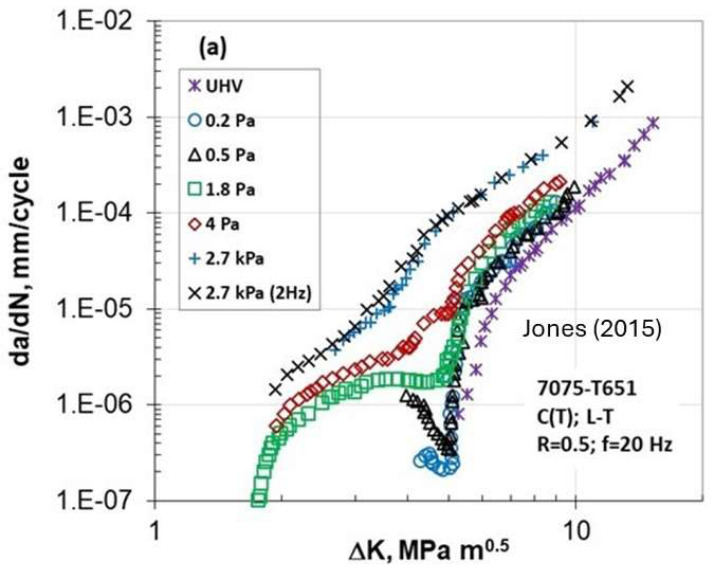

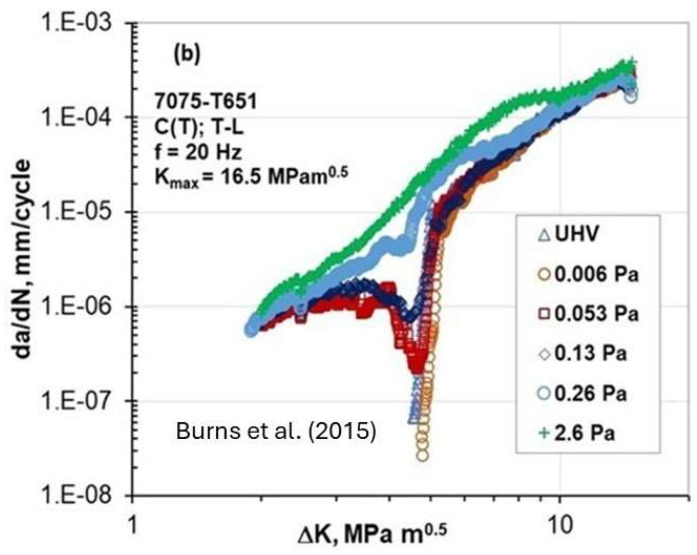
FCG behavior of 7075-T651 Al alloy at different water vapor pressures at (**a**) R-controlled and (**b**) K_max_-controlled tests [13,14].

More recently, Burns’ group from the University of Virginia [13,14] observed unexpected deep and acceleration behavior in da/dN versus ΔK for 7000 Al alloys. The material studied was a 50.8 mm thick 7075-T651 plate taken from a large-scale heat-treated plate to simulate a typical airframe fatigue. The microstructure was peak-aged, partially recrystallized with the grain size of 200–1000 μm at the plate center plane in the rolling-longitudinal (L) direction, 30–300 μm in the transverse (T) direction, and 10–70 μm in the thickness (S) direction. Tensile yield and ultimate strength in the longitudinal direction were 508 and 598 MPa, respectively. The plane strain fracture toughness was 33 MPa$\sqrt{m}$. Fatigue crack growth experiments were performed in accordance with ASTM E647, where crack length was calculated from crack mouth opening displacement using the compliance method. Compact tension (CT) specimens were machined in both the L–T and T–L orientations having a width and a thickness of 50.8 and 6.35 mm, respectively. Figure 7a shows da/dN vs. ΔK for (L-T) orientation at constant R = 0.5 and Figure 7b shows the K_max_ = 16.5 MPa$\sqrt{m}$ tests for (T-L) orientation.

Their tests were conducted in the ultra-high vacuum (UHV ~10^−6^ Pa), at different water vapor pressures, and in lab air. Interestingly, the (da/dN) data exhibit (da/dN)-ΔK curves with two different thresholds, one for vacuum and another for water vapor pressure greater than 1.8 Pa. The distinctive influence of water vapor pressure on abnormal FCG behavior is clearly seen in both R and K_max_ constant tests. In addition, two tests at R = 0.5 were conducted at a pressure of 2.7 kPa and at two frequencies, 2Hz and 20Hz. These two frequencies resulted in basically identical (da/dN)-ΔK behavior signifying no frequency effect. Their results show that below ΔK_th_ = 5 MPa$\sqrt{m}$, the Marci effect was observed, and this effect vanished at higher moisture content by approaching another threshold value lower than 2 MPa$\sqrt{m}$.

### 3.3. Observations in Steel

**Figure 8:**
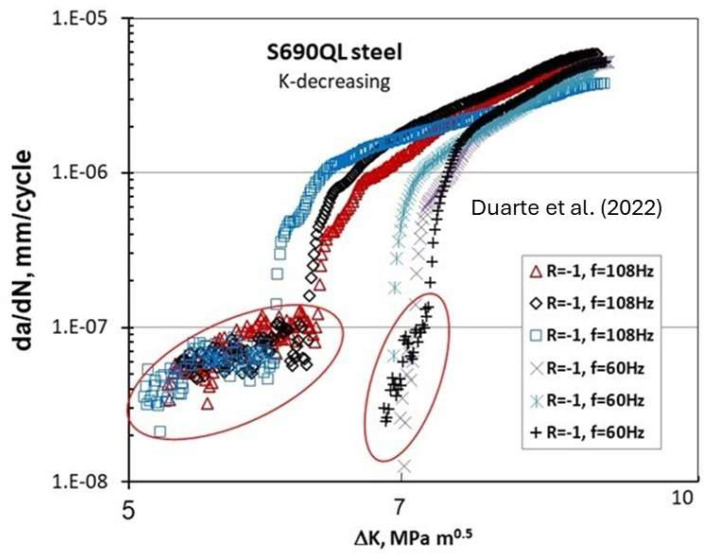
Effect of frequency on threshold and near-threshold behavior of S690QL steel at R = −1 at two frequencies, 108 Hz and 60 Hz [15].

Figure 8 depicts the FCG data of S690QL steel [15] (with the yield stress 810 MPa) at near-threshold for two frequencies, 108 Hz and 60 Hz, at R = −1.

The fatigue crack propagation tests were carried out using single edge notch bend (SENB) specimens with a width of 19 mm and a thickness of 6 mm. The specimen orientation was arranged so that the crack propagation plane was parallel to the rolling direction. An 8-point bending fixture was utilized for the tests, and crack growth was monitored using the direct current potential drop (DCPD) technique with temperature compensation. The fatigue crack propagation threshold tests were conducted using the *K*-decreasing (or load-shedding) technique, following the guidelines outlined in the test standard ASTM E647.

Deviation in the FCG behavior seen below the 10^−7^ mm/cycle indicates that design based on ΔK_th_ corresponding to the FCG rate of 10^−7^ mm/cycle would be nonconservative. The FCG behavior below 10^−7^ mm/cycle shows a much larger scatter/variation for 108 Hz than for 60 Hz.

**Figure 9:**
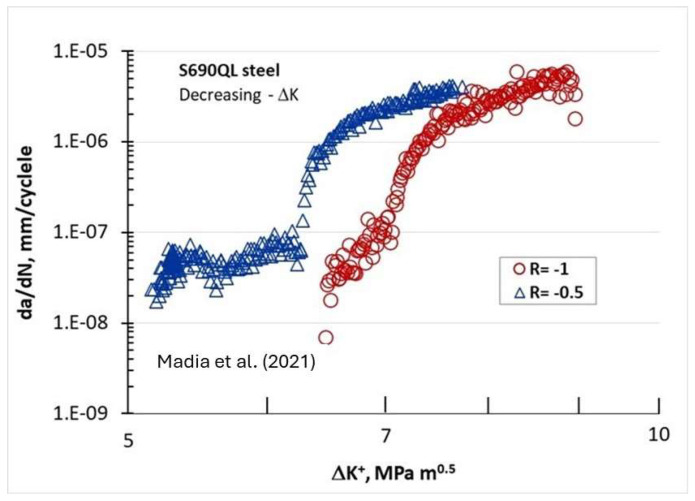
FCG behavior of S690QL steel in terms of (da/dN)-ΔK^+^ for two negative R-ratios of −0.5 and −1 [16].

Figure 9 shows the FCG behavior of the same S690QL steel [16] in terms of (da/dN)-ΔK^+^ for two negative R-ratios of −0.5 and −1. In both negative R-ratios, the Marci effect is seen for the (da/dN) < 10^−7^ mm/cycle. Interestingly, this behavior is observed even for negative R and is more apparent at the −0.5 than the −1 R-ratio.

The effect of humidity (in the laboratory air with relative humidity between 30% and 70%) on near-threshold FCG was also observed [15,16]. Transient FCG behavior followed an abrupt change in the humidity level, indicating that environmental damage accumulated in the crack tip. No pleasurable explanation for the observed transient FCG was provided.

## 4. Discussion

**Figure 10:**
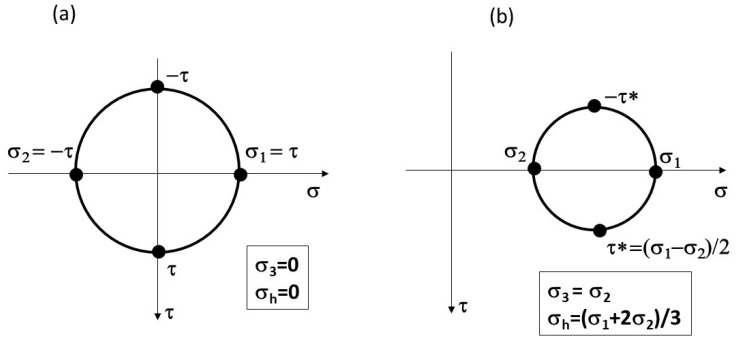
Illustration of hydrostatic stress condition for (**a**) planar slip and (**b**) wavy slip alloys.

As previously mentioned, the Ni superalloys, Ni100, Inconel 718 and Rene’95, that in general exhibit strong planar slip deformation did not show the Marci effect. These planar slip alloys may have a low hydrostatic stress, σ_h_, on their gliding plane. On the other hand, wavy slip alloys may encounter relatively high hydrostatic stress just ahead of the crack tip under plane strain conditions. Figure 10 illustrates the expected state of hydrostatic stress for pure shear (planar slip) versus plane strain (wavy slip) near the fatigue crack tip. It can be hypothesized that the “Marci effect” is linked with time-dependent void generation (due to cross slip) under plane strain conditions for wavy slip alloys due to the presence of high hydrostatic stress, σ_h_. Voids are commonly observed in tensile tests for ductile materials exhibiting necking due to the existence of a relatively high hydrostatic stress at the necking area. In creep, voids are observed due to high tensile stress. Hence, the wavy slip alloys may be more susceptible to the Marci effect due to the presence of the environment, which can assist further cross slip activity to stimulate time/rate-dependent room temperature void generation.

A close examination of Figure 4, Figure 5, Figure 6, Figure 7, Figure 8 and Figure 9 indicates three different types of abnormal FCG behavior linked presumably to the slip deformation and presence of the hydrostatic stress.

**Figure 11:**
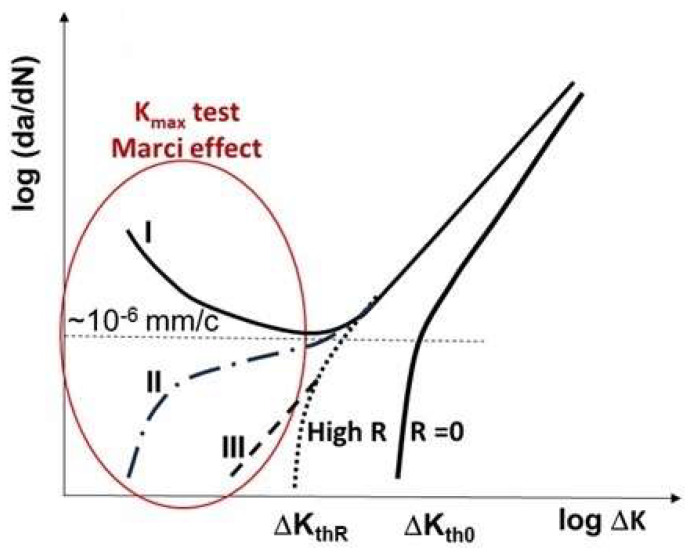
Illustration of three types of “Marci effect” observed at low ΔK applied.

Figure 11 illustrates these three types of “Marci effect” observed in an active environment, e.g., lab air. In the case of a vacuum, these three types are also present with reduced FCG rates in the range of 3–5 times (Figure 5 and Figure 6) as seen in lab air. For example, wavy slip Ti superalloys, MI834 and Ti-6246, exhibit a plateau and then acceleration in the FCG rate (type I behavior). However, 7075-T651 Al with mixed planar and wavy slip deformation exhibit a dip and subsequent acceleration, and then another dip approaching the second lower ΔK_th_ (type II). On the other hand, 8009Al (partially planar slip) and S690QL steel indicated the type III behavior. In the case of S690Ql steel, the change in (da/dN)-ΔK slope starts not at 10^−6^ but at 10^−7^ mm/cycle. This intrinsic process associated with the “Marci effect” is supplemented by the environment, which enhances cross slip activity and the generation of voids (in plane strain), causing faster FCG rate in air than under vacuum (Figure 5 and Figure 6). Possible explanations for Ti superalloys may be linked to their rate-(time-) dependent deformation related to room temperature creep and hold time effects on the FCG behavior [22], which may also play a role in the observed time-dependent “Marci effect”.

## 5. Conclusions

Based on the experimental results and reports in the literature on unusual FCG behavior of long cracks at low ΔK applied, the following conclusions can be drawn:

- The “Marci effect” is reported for several engineering alloys and showed sensitivity to partial water vapor pressure, frequency and material slip systems;
- Conventional application of linear elastic fracture mechanics to FCG analysis assumes that (da/dN) decreases monotonically with decreasing ΔK and approaches the threshold value of ΔK_th_ with the (da/dN) ≤ 10^−7^ mm/cycle;
- In contrast, some Ti, Al and steel alloys exhibit a dip and then an acceleration or plateau in (da/dN)-ΔK for long cracks tested at constant K_max_ or R-ratio conditions;
- This unusual (da/dN)-ΔK behavior is not comprehended and cannot be explained based on plasticity-, roughness- or oxide-induced crack closure (PICC, RICC or OICC) considerations.

An understanding of this unusual behavior represents a challenge to scientists and engineers. Overlooking or ignoring it may compromise fatigue safety of structures under high mean service loads. Hence, the primary aim of this article is to encourage the fatigue research community to further investigate the lack of traditional threshold and the unusual FCG behavior of long cracks at low ΔK < ΔK_th_ for specific engineering materials.

## Data Availability

Data are contained within the article.

## References

[1] (2015). Standard Test Method for Measurement of Fatigue Crack Growth Rates.

[2] (2018). Metallic Materials—Fatigue Testing—Fatigue Crack Growth Method.

[3] Paris P., Erdogan F. (1963). A Critical Analysis of Crack Propagation Laws. J. Basic. Eng..

[4] Elber W. (1971). The significance of fatigue crack closure. Damage Tolerance in Aircraft Structures ASTM STP.

[5] Suresh S. (2001). Fatigue of Materials.

[6] Pippan R., Hohenwater A. (2017). Fatigue crack closure: A review of the physical phenomena. Fatigue Fract. Eng. Mater. Struct..

[7] Kujawski D., Vasudevan A.K., Ricker R.E., Sadananda K. (2023). On 50 years of fatigue crack closure dispute. Fatigue Fract. Eng. Mater. Struct..

[8] Marci G. Failure mode below 390 K with IMI 834. Proceedings of the 6th International Fatigue Conference (FATIGUE 96).

[9] Lang M., Hartman G.A., Larsen J.M. (1998). Investigation of an abnormality in fatigue crack growth curves—The Marci Effect. Scr. Metall..

[10] Sarrazin-Baudoux C., Petit J., Mignot F., Docquet V. Investigation of the abnormal near threshold fatigue crack propagation on a Ti6246 alloy. Proceedings of the ECF 14: Fracture Mechanics beyond 2000: 14th European Conference on Fracture.

[11] Piascik R.S., Newman J.A. (2002). Accelerated Near-Threshold Fatigue Crack Growth Behavior of an Aluminum Powder Metallurgy Alloy.

[12] Newman J.A., Piascik R.S. (2003). Near Threshold Fatigue Crack Growth Behavior of Fine-Grain Nickel-Based Alloy.

[13] Burns J.T., Bush R.W., Jones J.L., Lee Y., Gangloff R.P. (2015). Effect of water vapor pressure on fatigue crack growth in Al–Zn–Cu–Mg over wide-range stress intensity factor loading. Eng. Fract. Mech..

[14] Jones J.L. (2015). The Effect of Water Vapor Pressure on the Fatigue Crack Propagation Rates in Aerospace Aluminum Alloys 7075-T651 and 2199-T86. Master’s Thesis.

[15] Duarte L., Schönherr J.A., Madia M., Zerbst U., Geilen M.B., Klein M., Oechsner M. (2022). Recent developments in the determination of fatigue crack propagation thresholds. Int. J. Fatigue.

[16] Madia M., Vojtek T., Duarte L., Zerbst U., Pokorný P., Jambor M., Hutar P. (2021). Determination of fatigue crack propagation thresholds for steel in presence of environmental effects. Int. J. Fatigue.

[17] Vasudevan A.K., Sadananda K., Lout N. (1992). Reconsideration of fatigue crack closure. Scr. Metall. Et Mater..

[18] Riemelmoser F.O., Pippan R. (1997). Investigation of a growing fatigue crack by means of a discrete dislocation model. Mater. Sci. Eng. A.

[19] Paris P.C., Tada H., Donald J.K. (1999). Service load fatigue damage—A historical perspective. Int. J. Fatigue.

[20] Vasudevan A.K., Sadananda K., Lout N. (1993). Two critical stress intensities for threshold fatigue crack propagation. Scr. Met. Mater..

[21] Vallellano C., Navarro A., Dominguez J. (2013). Two-parameter fatigue crack growth driving force: Successive blocking of the monotonic and cyclic plastic zones at microstructural barriers. Int. J. Fatigue.

[22] Evans W.J., Gostelow C.R. (1979). The effect of hold time on the fatigue properties of a b-processed titanium alloy. Met. Trans..

